# Positive childhood experiences and life satisfaction among Chinese college students: serial mediation through perceived social support and meaning in life

**DOI:** 10.3389/fpsyt.2026.1921967

**Published:** 2026-07-30

**Authors:** Yan Yang, Pan Chen, Zhenzhen Chen, Peng Lei

**Affiliations:** 1 School of Art, Southeast University, Nanjing, China; 2 School of Tourism and Cultural Industries, Sichuan Tourism University, Chengdu, China; 3 Institute of Economics, Shanghai Academy of Social Sciences, Shanghai, China

**Keywords:** Chinese college students, emerging adulthood, life satisfaction, meaning in life, perceived social support, positive childhood experiences, serial mediation

## Abstract

**Background:**

Positive childhood experiences (PCEs) constitute foundational environmental protective factors associated with long-term psychological well-being; however, the mechanisms linking early relational environments to life satisfaction (LS) among college students remain insufficiently understood. Grounded in Broaden-and-Build Theory and Conservation of Resources Theory, this study examined perceived social support (PSS) and meaning in life (ML) as sequential mediating resources in the association between PCEs and LS among Chinese undergraduates.

**Methods:**

A cross-sectional sample of 1,352 undergraduates (M_age = 19.37, SD = 1.06; 67.5% female) from four universities in Sichuan Province, China, completed validated measures of PCEs, PSS, ML, and LS. Serial mediation analysis was conducted using PROCESS Model 6 with 5,000 bootstrap resamples, controlling for seven demographic covariates; measurement validity checks and four sets of sensitivity analyses assessed the robustness of the results.

**Results:**

PCEs were positively associated with LS (completely standardized total effect = .414, p <.001). All three specific indirect pathways were significant: through PSS alone (completely standardized effect = .161, 95% CI [.113,.209]), through ML alone (.053, 95% CI [.031,.077]), and through PSS and ML sequentially (.062, 95% CI [.042,.085]), together accounting for 66.5% of the total association. The pathway through PSS was significantly larger than the other two, which were of comparable magnitude. The direct association remained significant (β = .139, p <.001), indicating partial mediation. The pattern of results was robust to the removal of PCE items overlapping in content with the PSS measure, to the separate specification of the two ML dimensions, and across genders.

**Conclusion:**

The findings are consistent with a sequentially organized resource pathway in which positive early relational experiences relate to life satisfaction through social and existential mechanisms. Pending longitudinal confirmation, strengthening perceived social support and fostering meaning in life represent complementary intervention targets for promoting well-being among Chinese college students.

## 1 Introduction

### 1.1 Research background and problem statement

Over the past two decades, research on childhood experiences and adult psychological outcomes has been dominated by the adverse childhood experiences (ACEs) framework, documenting how early exposure to abuse, neglect, and household dysfunction confers lasting developmental risks (1). While this work has generated invaluable insights into developmental vulnerability, its predominant deficit orientation has left a complementary question underexplored: what role do positive childhood experiences (PCEs) play in shaping long-term psychological well-being? A growing body of scholarship has begun to address this asymmetry, conceptualizing supportive early experiences not merely as the absence of adversity but as active contributors to healthy development (2, 3). A systematic review of 87 studies confirmed that higher PCE levels are consistently associated with decreased depressive symptoms, reduced anxiety, and improved subjective well-being across diverse adult populations (4). In a large population-based study of pandemic-era youth in British Columbia, Canada, Samji et al. (5) reported that PCEs showed protective associations with depression, mental well-being, and life satisfaction that were comparable to or exceeded the corresponding associations for ACEs in that sample, supporting the view that positive early experiences constitute an active resource base rather than merely the absence of adversity. This paradigm shift from deficit-oriented to resource-oriented frameworks carries important implications for both research and intervention design.

PCEs encompass a range of supportive interpersonal experiences during childhood and adolescence, including warm family relationships, peer support, the presence of caring non-parental adults, and engagement with community traditions (2). Latent profile analyses in Chinese young adults have identified distinct experience profiles; the High Benevolent/Low Adverse profile was associated with significantly better mental health outcomes and life satisfaction relative to profiles characterized by elevated adversity, underscoring the independent value of positive early experiences within Chinese developmental contexts (6). The cultural validity of the PCE framework in Chinese populations has been confirmed by Zhan et al. (7), who validated a PCE measure in a large community adult sample (N = 6,929), and by Yu et al. (8), who demonstrated its applicability in Chinese college students.

Life satisfaction (LS), defined as a global cognitive-evaluative appraisal of one’s life relative to personally held standards (9), represents a central indicator of subjective well-being across developmental stages and cultural contexts. Among Chinese college students, LS has attracted considerable research attention because this population navigates a transition marked by heightened academic demands, evolving social relationships, and increasing autonomy from family (10). The present study was conducted among undergraduates from four universities in Sichuan Province, a sample characterized by a high proportion of rural-origin students (68.6%) and modest economic backgrounds, providing a contextually grounded test of early experience effects in a population for whom relational resources may assume particular compensatory importance.

Despite growing evidence for the PCE–LS association, the psychological mechanisms through which early positive experiences relate to adult LS remain insufficiently understood. Prior research has established that PCEs are positively associated with broader mental health and subjective well-being indicators (2, 7), yet the specific intermediate processes, particularly the roles of perceived social support (PSS) and meaning in life (ML), have rarely been examined within an integrated theoretical framework. Clarifying these mediating pathways is theoretically important for understanding how early relational resources relate to adult well-being and practically important for identifying modifiable targets in college student interventions. The present study addresses this gap by examining a serial mediation model in which PSS and ML are proposed as sequential mechanisms linking PCEs to LS.

### 1.2 Theoretical framework and hypotheses development

The present study integrates two complementary theoretical frameworks. Fredrickson’s (11) Broaden-and-Build Theory posits that positive emotional experiences and enriching early environments broaden individuals’ momentary thought-action repertoires, and that these broadened states, over time, build enduring personal resources—including social, cognitive, and psychological assets—that persist beyond the transient experiences that generated them. PCEs spanning family, peer, and community domains represent precisely the sustained positive interpersonal environment this theory identifies as conducive to durable resource accumulation. Hobfoll’s (12) Conservation of Resources (COR) theory provides a complementary logic: individuals strive to acquire and accumulate valued psychological resources, and those who begin adulthood with a richer initial resource base are better positioned to consolidate additional resources over time. Within this framework, perceived social support functions as a proximal social resource cultivated through early relational experiences, while meaning in life represents a higher-order existential resource that depends, in part, on the relational foundation social support provides.

#### 1.2.1 Positive childhood experiences and life satisfaction

Attachment theory provides a developmental foundation for understanding how PCEs may be associated with adult LS. Bowlby’s (13) model holds that children who experience consistent warmth, responsiveness, and protection develop secure internal working models, stable cognitive-affective representations of the self as worthy of care and of others as reliable, that persist into adulthood and shape appraisals of the social environment and global life evaluation. PCEs encompassing family support, peer connectedness, and community belonging may thereby establish a relational foundation associated with positive self-appraisal and favorable life evaluations. Bethell et al. (2) documented significant dose-response associations between cumulative PCEs and adult mental health indicators, and related work has linked positive early family and social experiences to higher subjective well-being in adult populations (14). Among Chinese young adults, higher cumulative PCE scores have been associated with attenuation of the negative relationship between ACEs and adult flourishing, with protective associations particularly pronounced under conditions of cumulative adversity exposure (15). A prospective study further demonstrated that ML was the strongest predictor of stable LS trajectories among individuals with histories of adverse childhood exposure, underscoring the developmental importance of experiential resources cultivated through early positive environments (16). We therefore propose:

H1: Positive childhood experiences are positively associated with life satisfaction among Chinese college students.

#### 1.2.2 The mediating role of perceived social support

Two processes are proposed to link PCEs to PSS. First, early experiences of family responsiveness, peer support, and meaningful mentorship may be internalized as generalized positive relational expectations, cognitive templates through which individuals in adulthood perceive social signals of support and care (13, 17). Individuals with richer positive relational histories may perceive available support more readily, not because their objective networks are necessarily larger, but because their relational schemas facilitate more favorable appraisals of social availability. Second, consistent with COR theory, early positive relational experiences may directly cultivate the social competencies and network ties that underlie perceived support availability in adulthood (12).

The association between PSS and LS is among the most consistently replicated findings in well-being research (10). Social support benefits well-being through both a stress-buffering mechanism and a direct main-effects pathway whereby the subjective sense of being supported independently contributes to positive life evaluations (18). A systematic review encompassing 51 studies confirmed that PSS exerts both direct positive associations with emotional well-being and indirect associations via resilience and LS among college students (19). A chain mediation study further demonstrated that PSS positively predicted LS among college students through sequential pathways involving physical activity and self-control, highlighting the multifaceted functional role of social support in well-being processes (20). Among Chinese adults, Yang et al. (21) found that PSS and psychological resilience jointly mediated the negative association between ACEs and LS, positioning PSS as a critical resource variable in early-experience-to-well-being pathways. We therefore hypothesize:

H2: Perceived social support mediates the positive association between positive childhood experiences and life satisfaction (PCEs →PSS → LS).

#### 1.2.3 The mediating role of meaning in life

ML, defined as the subjective sense that one’s existence is purposeful, significant, and coherent (22), has been characterized as a fundamental psychological need whose fulfillment is essential for sustained well-being (23). Self-determination theory situates ML as emerging from satisfaction of basic psychological needs for autonomy, competence, and relatedness (24), suggesting that early experiences of genuine relational support and community belonging may lay the experiential groundwork for existential purpose.

PCEs may be associated with ML through two pathways. Experiences of family warmth, peer acceptance, and mentorship provide children with early encounters of being valued and contributing to something beyond themselves, experiences Frankl (23) identifies as foundational to meaning construction. Additionally, community participation and school belonging during adolescence may foster a sense of connectedness to a larger social fabric, constituting a primary source of existential meaning (25). A chain mediation study among Chinese adolescents found that perceived stress was negatively associated with LS through sequential reductions in self-efficacy and ML, positioning ML as a particularly proximal resource linking psychological states to well-being evaluations (26). Across a cross-cultural sample, Yıldırım et al. (27) demonstrated that ML constituted a significant mediator between adverse psychological experiences and distress, with effect sizes exceeding those of other psychological resources including resilience and PSS. The association between ML and LS is well-established: individuals who experience their lives as purposeful and coherent report substantially higher global life evaluations (28, 29), and a recent meta-analysis confirmed medium-to-strong positive associations between ML and positive mental health indicators among college students (30). We therefore hypothesize:

H3: Meaning in life mediates the positive association between positive childhood experiences and life satisfaction (PCEs → ML →LS).

#### 1.2.4 Serial mediation: from perceived social support to meaning in life

Beyond functioning as parallel independent mediators, PSS and ML may operate in a sequential chain in which social support serves as a relational antecedent to meaning construction. The subjective experience of being reliably supported and valued by significant others constitutes in itself a relational source of existential meaning (25). When individuals perceive themselves as embedded in supportive social networks, this sense of social embeddedness affirms that one matters to others and belongs to something larger than oneself, experiences central to a meaningful life (31). This account is particularly relevant within the Chinese cultural context, where meaning-making is deeply relational in character and the sense of occupying a valued role within an interpersonal network constitutes a primary source of existential purpose (32).

This cultural-contextual rationale merits elaboration. Within the Confucian relational tradition, the self is constituted relationally rather than atomistically: individuals understand who they are largely through their positions within webs of reciprocal relationships, an orientation captured by the construct of interdependent self-construal (33). Under this cultural ontology, existential significance is not primarily an individual achievement of self-defined purpose but is intrinsically anchored in occupying, and adequately fulfilling, a valued role within one’s relational network—as a family member, friend, student, and future contributor to society. The perception of being reliably supported therefore carries a meaning-relevant significance that extends beyond its instrumental coping function: to perceive oneself as supported is to receive ongoing confirmation of one’s valued standing within the relational order, and this confirmation is itself constitutive of a meaningful existence. From this perspective, the hypothesized PSS → ML pathway in Chinese samples reflects a culturally grounded constitutive relation rather than a merely contingent empirical association between two independent resources. This reasoning provides a stronger *a priori* rationale for the serial specification in the present cultural context, while simultaneously raising the question—addressed in the concluding discussion—of whether the same mediation architecture would be reproduced in individualistic cultural settings, where meaning-making is more strongly tethered to autonomous goal pursuit.

Empirical support for the PSS → ML association has been substantially strengthened by recent evidence. A meta-analysis of 135 studies and 397 effect sizes confirmed a significant positive correlation between social support and ML across multiple populations, with subjective social support demonstrating the strongest association (34). A chain mediation study among Chinese university students found that social support was negatively associated with maladaptive disengagement behavior through sequential mediation involving ML and psychological resilience, with the full chain accounting for 70.2% of the total effect, providing direct evidence for PSS as an upstream predictor of ML in this population (35). Tian et al. (36) further documented longitudinally that PSS predicted lower depression among college students through sequential mediation of gratitude and ML, offering prospective evidence for ML as a downstream outcome of social support processes. Among Chinese adolescents, Yang et al. (37) confirmed that PSS was associated with greater ML through reduced psychological insecurity and enhanced hope, optimism, and self-efficacy. Converging evidence has consistently demonstrated that PSS positively predicts ML among Chinese college students (38, 39). We therefore propose:

H4: Perceived social support and meaning in life sequentially mediate the positive association between positive childhood experiences and life satisfaction (PCEs → PSS → ML → LS).

### 1.3 The present study

**Figure 1:**
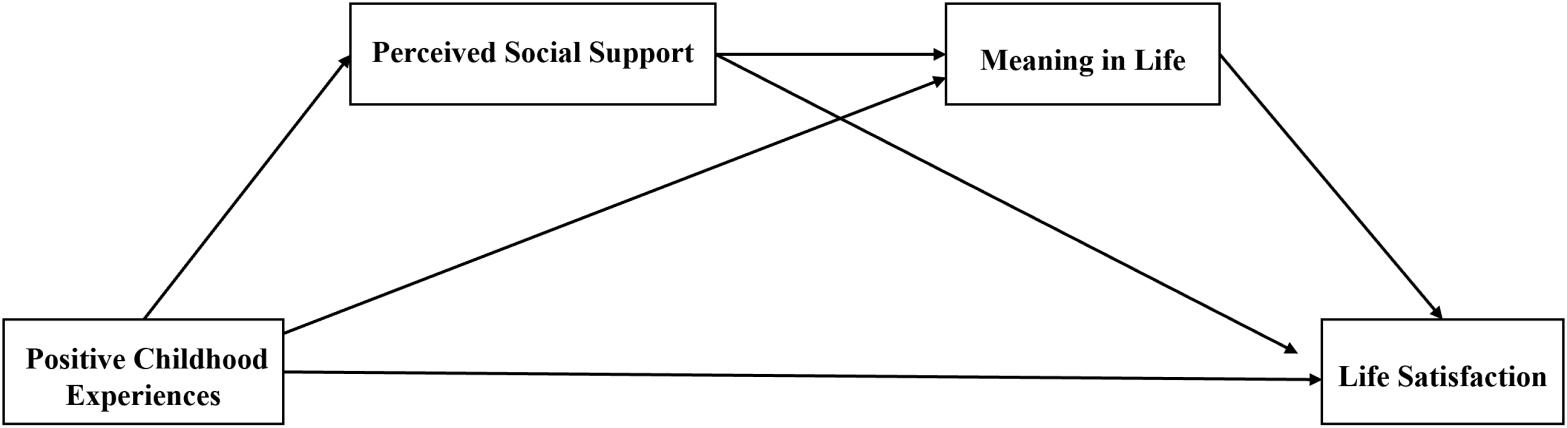
The proposed theoretical model.

The present study examined a serial multiple mediation model in which perceived social support and meaning in life were proposed to function as sequential psychological mechanisms linking positive childhood experiences to life satisfaction among Chinese college students. Using a sample of 1,352 undergraduates recruited from four universities in Sichuan Province and applying PROCESS Model 6 with 5,000 bootstrap resamples (40), the study estimated three specific indirect pathways alongside the direct association while controlling for key demographic variables. The proposed theoretical model is depicted in **Figure 1**.

This investigation contributes to the literature in three respects. First, whereas prior mediation research in the PCE literature has predominantly employed single-mediator or parallel-mediation designs, the present study is, to our knowledge, among the first to test the sequential organization of a social resource (perceived social support) and an existential resource (meaning in life) within the PCE–life satisfaction framework—thereby treating the structural relation between the two resource classes, rather than merely their individual mediating status, as an object of empirical testing. The design further incorporates formal pairwise contrasts among the three specific indirect pathways, moving beyond the question of whether each mechanism is statistically significant to a quantitative ordering of their relative magnitudes. Second, it integrates social resource accumulation and existential meaning construction within a unified sequential framework grounded jointly in Broaden-and-Build and Conservation of Resources theories, and motivates this specification with a culturally grounded account of relational meaning-making in the Chinese context. Third, by focusing on non-elite universities in an inland Chinese province with a high proportion of rural-origin and economically modest students, the study contributes contextually grounded evidence to a literature that has disproportionately relied on samples from affluent urban populations.

## 2 Methods

### 2.1 Participants and procedure

Participants were recruited from four universities in Sichuan Province, southwestern China, representing research-oriented, applied-oriented, and vocational institutional tiers. Data were collected between April and May 2026 via Wenjuanxing, a widely used Chinese online survey platform. A convenience sampling procedure was employed: recruitment notices were distributed through university student affairs offices, and students voluntarily self-selected into the study by completing the online survey. Because the notices were disseminated through institutional channels whose precise reach could not be determined, an exact invitation-based response rate could not be computed. All participants reviewed and electronically acknowledged an informed consent statement prior to survey completion. All procedures involving human participants were conducted in accordance with the ethical standards of the Declaration of Helsinki (41) and approved by the Ethics Review Sub-committee of the Sichuan Provincial Psychological Association.

Two attention-check items were embedded to identify inattentive responding. Of the 1,400 responses initially collected, 6 were excluded for incomplete responding, 32 for implausibly short completion times (mean response time below two seconds per item), and 10 for failing at least one attention-check item, yielding 1,352 valid questionnaires (effective retention rate = 96.6%). The four universities contributed 356, 372, 318, and 306 participants, respectively. All participants were first-year (n = 820, 60.7%) or second-year (n = 532, 39.3%) undergraduates, enrolled in natural sciences (57.5%) or social sciences (42.5%) fields. Detailed demographic characteristics are presented in **Table 1**.

**Table 1:** Demographic characteristics of the sample (N = 1,352).

Variable	Category	*n*	%
Gender	Male	439	32.5
	Female	913	67.5
Age (years)	18	284	21.0
(*M* = 19.37, *SD* = 1.06)	19	538	39.8
	20	334	24.7
	21	136	10.1
	22	60	4.4
Household registration	Urban	424	31.4
	Rural	928	68.6
Number of siblings	0 (only child)	326	24.1
	1	646	47.8
	2	255	18.9
	3	95	7.0
	≥ 4	30	2.2
Paternal education	High school or below	1,071	79.2
	Associate degree	126	9.3
	Bachelor's degree	151	11.2
	Master's or above	4	0.3
Maternal education	High school or below	1,111	82.2
	Associate degree	123	9.1
	Bachelor's degree	106	7.8
	Master's or above	12	0.9
Family annual income	< 50,000 RMB	486	35.9
	50,000–99,999 RMB	469	34.7
	100,000–199,999 RMB	270	20.0
	200,000–299,999 RMB	73	5.4
	≥ 300,000 RMB	54	4.0
University	University A	356	26.3
	University B	372	27.5
	University C	318	23.5
	University D	306	22.6
Year of study	Freshman	820	60.7
	Sophomore	532	39.3
Field of study	Natural sciences	778	57.5
	Social sciences	574	42.5

### 2.2 Measures

All instruments had been previously validated in Chinese-speaking populations and were administered using five-point Likert response formats unless otherwise noted. Higher scores on all scales reflect greater levels of the respective construct.

#### 2.2.1 Positive childhood experiences

Positive childhood experiences (PCEs) were assessed using the seven-item PCEs scale (2), which captures retrospective perceptions of supportive interpersonal experiences across three domains: family relationships, social connectedness, and community engagement. The Chinese adaptation was informed by the validation procedures of Zhan et al. (7), who established satisfactory reliability and validity of a related PCEs measure in a large Chinese community adult sample (N = 6,929; Cronbach’s α = .70), with further psychometric support for Chinese college student samples provided by Yu et al. (8). The original binary response format was adapted to a five-point Likert scale (1 = *never* to 5 = *always*) to enable finer-grained measurement of perceived experience intensity (8). Scores were computed as item means, with higher scores reflecting greater levels of PCEs. Cronbach’s α = .910 in the present sample. In the present sample, confirmatory factor analysis of the adapted five-point scale supported a unidimensional structure with all standardized loadings significant (range:.57–.81, all p <.001) and satisfactory comparative fit (CFI = .911, SRMR = .049); the RMSEA (.144) was elevated, a documented tendency of this index in low-degree-of-freedom models estimated in large samples (42), whereas the SRMR—which is robust to this artifact—indicated close fit (**Table 2**).

**Table 2:** Confirmatory factor analysis fit indices for study measures (N = 1,352).

Scale	Model	χ²	df	CFI	TLI	RMSEA	SRMR	Loadings
PCEs	Single-factor	409.12	14	.911	.867	.144	.049	.57–.81
MSPSS	Three-factor	811.25	51	.941	.924	.105	.040	.68–.90
MLQ	Two-factor	650.03	34	.934	.913	.116	.060	.76–.91
SWLS	Single-factor	57.67	5	.984	.969	.088	.022	.58–.84

N = 1,352. All loadings significant at p <.001 unless otherwise noted.

#### 2.2.2 Perceived social support

Perceived social support (PSS) was measured using the 12-item Multidimensional Scale of Perceived Social Support (MSPSS; 43), comprising three four-item subscales assessing support from significant others, family, and friends. The Chinese version developed by Yang et al. (44) incorporates a culturally informed adaptation of the significant other subscale, replacing “a special person” with “a special person (such as my teacher)” to better reflect the relational context of Chinese college students. Yang et al. (44) validated this version in a sample of 2,830 university students (CFI = .98, RMSEA = .079; Cronbach’s α = .906; four-week test-retest reliability = .914). Items were rated on a five-point scale (1 = *strongly disagree* to 5 = *strongly agree*), and scores were computed as item means. Cronbach’s α = .956 in the present sample. In the present sample, confirmatory factor analysis supported the three-factor structure (CFI = .941, TLI = .924, RMSEA = .105, SRMR = .040), with standardized loadings ranging from.68 to.90 (all p <.001; **Table 2**).

#### 2.2.3 Meaning in life

Meaning in life (ML) was assessed using the 10-item Meaning in Life Questionnaire (MLQ; 22), comprising two five-item subscales: Presence of Meaning (the degree to which life is currently experienced as meaningful) and Search for Meaning (the degree to which one actively pursues meaning), with one Presence item reverse-scored. The Chinese version validated by Luo et al. (45) was used, which demonstrated strict longitudinal measurement invariance and a robust two-factor structure across a one-year four-wave study of Chinese college students (CFI ≥.92, RMSEA ≤.08; subscale α = .78–.86). The original seven-point format was adapted to a five-point Likert scale (1 = *absolutely untrue* to 5 = *absolutely true*) for consistency across instruments. In the present mediation model, ML was operationalized as the composite of the two subscale means (theoretical range: 2–10), reflecting its conceptualization as an integrated psychological resource consistent with the Conservation of Resources framework, and Cronbach’s α = .921 in the present sample. In the present sample, the Presence and Search subscales were substantially and positively correlated (r = .716), consistent with cross-cultural evidence that in interdependent cultural contexts searching for meaning does not connote a deficit of meaning and relates positively—rather than negatively, as typically observed in Western samples—to both presence of meaning and well-being (46). Confirmatory factor analysis supported the two-factor structure (CFI = .934, TLI = .913, RMSEA = .116, SRMR = .060), with standardized loadings ranging from.76 to.91 for all items (all *p* <.001; **Table 2**).

#### 2.2.4 Life satisfaction

Life satisfaction (LS) was assessed using the five-item Satisfaction with Life Scale (SWLS; 9), designed to measure the cognitive-evaluative component of subjective well-being. The Chinese version validated by Bai et al. (47) was employed, using a nationally representative mainland Chinese sample of 4,795 adults from all 31 provinces, which confirmed acceptable single-factor model fit (CFI = .99, RMSEA = .085; Cronbach’s α = .88) and strict measurement invariance across gender. The original seven-point format was adapted to a five-point Likert scale (1 = *strongly disagree* to 5 = *strongly agree*), with scores computed as item means (range: 1–5). Cronbach’s α = .907 in the present sample. In the present sample, confirmatory factor analysis supported the single-factor structure (CFI = .984, TLI = .969, RMSEA = .088, SRMR = .022), with standardized loadings ranging from.58 to.84 (all p <.001; **Table 2**).

### 2.3 Data analysis strategy

All analyses were conducted using SPSS Statistics and the PROCESS macro version 4.2 (40). In the preliminary stage, descriptive statistics and bivariate Pearson correlations were computed for all core study variables. Common method bias was assessed using Harman’s single-factor test (48); the first unrotated factor accounted for 24.57% of total variance, well below the conventional 40% threshold, suggesting that common method bias was unlikely to seriously distort the observed inter-construct associations. Given the recognized limitations of Harman’s test as a single-index diagnostic, this result is treated as a preliminary rather than definitive indicator.

In the primary stage, a serial multiple mediation model was estimated using PROCESS Model 6. PCEs were specified as the independent variable (X), PSS as the first mediator (M_1_), ML as the second mediator (M_2_), and LS as the dependent variable (Y). Seven demographic variables were included as covariates in all model equations: gender, age, household registration type, number of siblings, paternal educational level, maternal educational level, and family annual income. The significance of indirect effects was evaluated using bias-corrected bootstrap confidence intervals based on 5,000 resamples, with significance indicated by 95% confidence intervals excluding zero (40). Three specific indirect pathways were examined: Ind1 (PCEs → PSS → LS), Ind2 (PCEs → ML → LS), and Ind3 (PCEs → PSS → ML → LS). Pairwise contrasts (C1: Ind1 vs. Ind2; C2: Ind1 vs. Ind3; C3: Ind2 vs. Ind3) were additionally tested to evaluate the relative magnitude of each pathway.

The hypothesized four-factor measurement model fit the data significantly better than a three-factor alternative merging PCEs and PSS into a single factor (Δχ²(3) = 724.30, *p* <.001; ΔCFI = .044) and a single-factor model loading all items on one factor (Δχ²(6) = 2,754.64, *p* <.001), with information criteria (AIC, BIC) likewise favoring the four-factor solution (**Table 3A**). The severely degraded fit of the single-factor model (CFI = .547, SRMR = .114) further corroborates, beyond Harman’s test, that common method variance is unlikely to account for the observed associations. Given the ordered-categorical response format, the four-factor model was re-estimated with the WLSMV estimator, yielding acceptable fit (CFI = .922, RMSEA = .066, SRMR = .056; TLI = .885). Notably, the latent correlation between PCEs and PSS (φ = .738) remained below the square roots of the AVE for both constructs (.762 and.796), satisfying the Fornell-Larcker criterion at the latent level—an evidentiary standard more stringent than the observed-correlation comparison—and converging with the HTMT result (.735; **Table 3B**).

**Table 3:** Measurement model evaluation: competing model comparisons and construct validity indices (N = 1,352).

Panel A. fit indices for competing measurement models											
Model	χ²	df		χ²/df	CFI	TLI	RMSEA	SRMR	AIC	BIC	Δχ² (Δdf) vs. four-factor
Four-factor (PCEs, PSS, ML, LS)	7,338.58	521		14.09	.922	.885	.066	.056	103,598.8	104,161.4	—
Three-factor (PCEs + PSS merged)	8,834.93	524		16.86	.756	.739	.108	.072	111,089.2	111,636.1	724.30 (3)***
Single-factor (all items)	15,945.56	527		30.26	.547	.518	.147	.114	118,193.8	118,725.2	2,754.64 (6)***
Panel B. convergent and discriminant validity indices											
Construct	CR		AVE				1		2	3	4
1. PCEs	.906		.581				**.762**		.735	.507	.502
2. PSS	.950		.633				.673		**.796**	.535	.516
3. ML	.927		.577				.456		.505	**.760**	.506
4. LS	.908		.667				.437		.473	.452	**.817**

MLQ items were specified on a single factor corresponding to the composite operationalization used in the mediation analysis. The four-factor model was superior to both alternatives on all criteria: chi-square difference tests (both p <.001), comparative fit (ΔCFI = .044 vs. three-factor, exceeding the.01 benchmark), and information criteria (lowest AIC and BIC). The severely degraded fit of the single-factor model (CFI = .547, SRMR = .114) further indicates that common method variance cannot account for the observed inter-construct associations. *** p <.001.

CR, composite reliability; AVE, average variance extracted. Bold diagonal values are the square root of AVE. Values below the diagonal are Pearson inter-construct correlations (all p <.001); values above the diagonal are HTMT ratios. Discriminant validity is supported when √AVE exceeds all correlations in the corresponding row and column (49) and when HTMT <.85 (50).

## 3 Results

### 3.1 Preliminary analyses

Descriptive statistics and bivariate correlations among all study variables are presented in **Table 4**. The four core variables were positively and significantly intercorrelated (all *p* <.001), with patterns consistent with the hypothesized model. PCEs demonstrated the strongest bivariate association with PSS (*r* = .673), followed by associations with ML (*r* = .456) and LS (*r* = .437). PSS and ML were moderately correlated (*r* = .505), and both were positively associated with LS (*r* = .473 and *r* = .452, respectively). These patterns provide preliminary support for the proposed mediating pathways and establish the empirical foundation for the serial mediation analysis.

**Table 4:** Descriptive statistics and bivariate correlations among study variables (N = 1,352).

Variable	M	SD	1	2	3	4	5	6
1. PCEs	3.46	0.87	—					
2. PSS	3.56	0.86	.673**	—				
3. ML	6.85	1.52	.456**	.505**	—			
4. PM	3.28	0.79	.481**	.529**	.916**^a^	—		
5. SM	3.57	0.84	.367**	.410**	.925**^a^	.716**	—	
6. LS	3.01	0.82	.437**	.473**	.452**	.506**	.336**	—

PCEs, Positive Childhood Experiences; PSS, Perceived Social Support; ML, Meaning in Life (composite score, range 2–10); PM, Presence of Meaning (range 1–5); SM, Search for Meaning (range 1–5); LS, Life Satisfaction. **p <.001. ^a^Correlations between ML and its component subscales are structurally determined by the composite operationalization (ML = PM mean + SM mean) rather than independently estimated.

**Table 4** also presents correlations for the Presence of Meaning (PM) and Search for Meaning (SM) subscales. PM and SM were substantially and positively correlated (*r* = .716), consistent with the cultural pattern discussed in Section 2.2.3. Both subscales were positively associated with LS, with PM showing a stronger association (*r* = .506) than SM (*r* = .336)—a pattern corroborated by the sensitivity analyses reported in Section 3.3.

### 3.2 Serial mediation analysis

**Figure 2:**
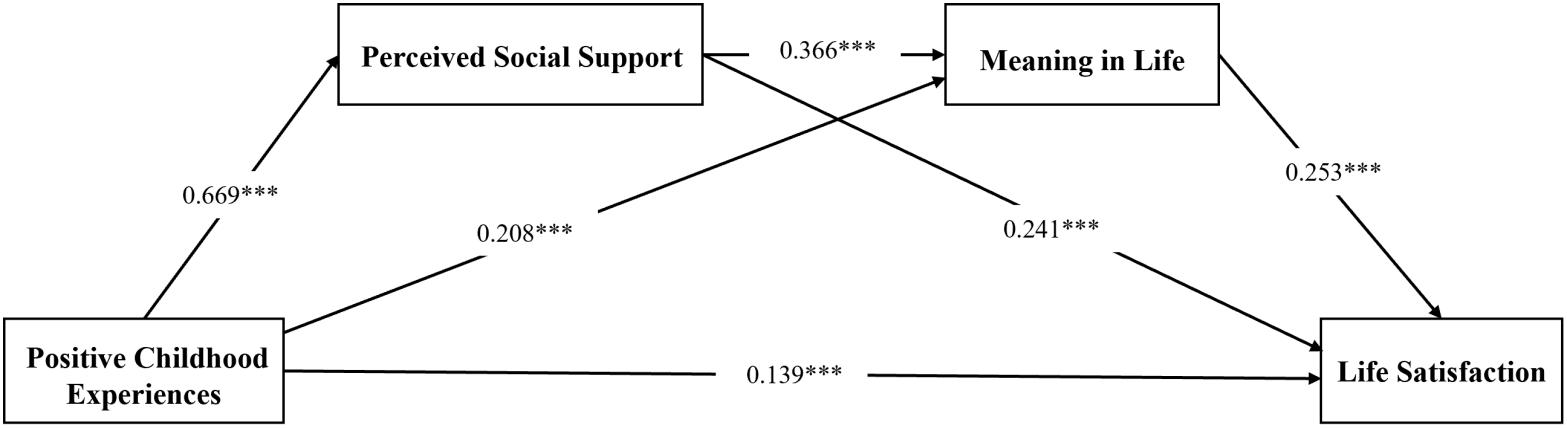
The final serial mediation model with standardized path coefficients. All paths controlled for gender, age, household registration type, number of siblings, paternal educational level, maternal educational level, and family annual income. ***p <.001.

It should be noted at the outset that the serial mediation model tests a statistical pattern consistent with, but not demonstrative of, the hypothesized temporal ordering among the variables. Results of the serial mediation model (PROCESS Model 6) are summarized in **Tables 5**, **6** and depicted in **Figure 2**. Seven demographic variables (gender, age, household registration type, number of siblings, paternal educational level, maternal educational level, and family annual income) were included as covariates in all model equations.

**Table 5:** Regression path coefficients in the serial mediation model.

Predictor	B	SE	t	β	VIF	R²	F
Outcome: PSS						.471	149.22***
Constant	.340	.359	0.95	—	—		
PCEs	.662	.020	32.95***	.669	1.05		
Outcome: ML						.283	58.92***
Constant	3.131	.739	4.24***	—	—		
PCEs	.363	.056	6.55***	.208	1.89		
PSS	.646	.056	11.52***	.366	1.89		
Outcome: LS						.321	63.24***
Constant	.796	.391	2.04*	—	—		
PCEs	.131	.030	4.41***	.139	1.95		
PSS	.229	.031	7.42***	.241	2.08		
ML	.137	.014	9.52***	.253	1.40		

PCEs, Positive Childhood Experiences; PSS, Perceived Social Support; ML, Meaning in Life; LS, Life Satisfaction. β represents completely standardized coefficients; standardized intercepts are zero by definition. All models controlled for gender, age, household registration type, number of siblings, paternal educational level, maternal educational level, and family annual income; covariate VIFs ranged from 1.02 to 2.08, and all VIF values were well below the conventional threshold of 5. Paternal and maternal educational levels showed significant coefficients of opposing signs in the PSS equation (β = .107 and β = −.125, respectively); as their VIF values (2.06 and 2.00) rule out severe multicollinearity, this pattern most plausibly reflects mutual suppression between the two highly correlated indicators. *p <.05, ***p <.001.

**Table 6:** Total, direct, and indirect effects of PCEs on life satisfaction.

Effect	*B*	*SE*	*t*	*p*	95% CI
Total effect					
PCEs → LS	.391	.023	16.76	<.001	[.345,.436]
Direct effect					
PCEs → LS	.131	.030	4.41	<.001	[.073,.189]
Indirect effects	B	Boot SE			Boot 95% CI
Total indirect	.260	.024	—	—	[.214,.306]
Ind1: PCEs → PSS → LS	.152	.024	—	—	[.106,.196]
Ind2: PCEs → ML → LS	.050	.011	—	—	[.030,.073]
Ind3: PCEs → PSS → ML → LS	.058	.010	—	—	[.040,.080]
Pairwise contrasts					
C1: Ind1 − Ind2	.102	.029	—	—	[.045,.156]
C2: Ind1 − Ind3	.093	.028	—	—	[.038,.146]
C3: Ind2 − Ind3	−.009	.014	—	—	[−.036,.019]

Bootstrap sample size = 5,000. 95% confidence intervals for indirect effects are bias-corrected percentile intervals. Completely standardized total indirect effect = .276, 95% CI [.227,.324]; Ind1 = .161, 95% CI [.113,.209]; Ind2 = .053, 95% CI [.031,.077]; Ind3 = .062, 95% CI [.042,.085]. A confidence interval excluding zero indicates a statistically significant effect. PCEs, Positive Childhood Experiences; PSS, perceived social support; ML, meaning in life; LS, life satisfaction.

As shown in **Table 5**, PCEs were significantly and positively associated with perceived social support (β = .669, *t* = 32.95, *p* <.001), accounting for 47.1% of variance in PSS. Among the covariates, gender (β = .099, *p* <.001), age (β = .041, *p* = .045), paternal educational level (β = .107, *p* <.001), and maternal educational level (β = −.125, *p* <.001) were significantly associated with PSS; the opposing signs of the two parental education coefficients most plausibly reflect mutual suppression between these highly correlated covariates (see **Table 5** footnote) and are not interpreted substantively.

When both PCEs and PSS were associated with meaning in life, both variables contributed significantly: PCEs (β = .208, *t* = 6.55, *p* <.001) and PSS (β = .366, *t* = 11.52, *p* <.001) jointly accounted for 28.3% of variance in ML. The stronger standardized coefficient for PSS relative to PCEs in this equation indicates that perceived social support was more strongly associated with meaning in life than were positive childhood experiences when the two were considered simultaneously. None of the seven demographic covariates reached conventional significance in this equation (all *p* >.05).

In the full model predicting life satisfaction, all three key associations were significant: PCEs (β = .139, *t* = 4.41, *p* <.001), PSS (β = .241, *t* = 7.42, *p* <.001), and ML (β = .253, *t* = 9.52, *p* <.001), collectively explaining 32.1% of variance in LS. Among the covariates, gender (β = −.060, *p* = .009), number of siblings (β = −.067, *p* = .006), and family annual income (β = .107, *p* <.001) were significantly associated with LS.

As shown in **Table 6**, the total association of PCEs with life satisfaction was significant and positive (β = .414, *B* = .391, *SE* = .023, *t* = 16.76, *p* <.001). After controlling for both mediators, the direct association of PCEs with LS remained statistically significant but was substantially attenuated (β = .139, *B* = .131, *SE* = .030, *t* = 4.41, *p* <.001), indicating partial mediation. The direct association accounted for approximately 33.5% of the total association, with the remaining 66.5% statistically accounted for by the two mediators.

The total indirect effect was significant (completely standardized effect = .276, 95% CI [.227,.324]). All three specific indirect pathways were individually significant. Ind1, representing the independent mediation of perceived social support (PCEs → PSS →LS), yielded the largest specific indirect effect (completely standardized effect = .161, 95% CI [.113,.209]), accounting for approximately 38.9% of the total association. Ind3, the serial pathway through both mediators (PCEs → PSS → ML →LS), was also significant (completely standardized effect = .062, 95% CI [.042,.085]), accounting for approximately 14.9% of the total association and consistent with the hypothesized sequential pathway from perceived social support to meaning in life. Ind2, representing the independent mediation of meaning in life (PCEs → ML →LS), was likewise significant (completely standardized effect = .053, 95% CI [.031,.077]), accounting for approximately 12.7% of the total association.

Pairwise contrasts among the three specific indirect effects revealed that Ind1 was significantly larger than both Ind2 (C1: *B* = .102, 95% CI [.045,.156]) and Ind3 (C2: *B* = .093, 95% CI [.038,.146]). By contrast, the difference between Ind2 and Ind3 did not reach statistical significance (C3: *B* = −.009, 95% CI [−.036,.019]), indicating that the independent mediation of meaning in life and the serial mediation pathway were of comparable magnitude. Standardized path coefficients for all model pathways are presented in **Figure 2**.

### 3.3 Sensitivity analyses

Four sets of sensitivity analyses were conducted to assess the robustness of the mediation results. First, to address potential content overlap between the PCEs and PSS measures, the model was re-estimated using a reduced PCEs score (PCE-R) that excluded the three items directly mirroring the MSPSS family and friends subscales (see **Table 7** for full results). The reduced scale correlated less strongly with PSS (*r* = .631 vs. *r* = .673 for the full scale), yet all three indirect pathways remained significant [Ind1 = .149, 95% CI (.105,.194); Ind2 = .052, 95% CI (.031,.075); Ind3 = .058, 95% CI (.039,.079)], with the pattern of results substantively unchanged. This indicates that the observed mediation structure is not an artifact of item content overlap.

**Table 7:** Serial mediation results with PCE-R as the independent variable (N = 1,352).

Effect	B	Boot SE	β (cs)	95% CI (cs)
Total effect	.0918***	.0055	.4124	[.3448,.4361]^a^
Direct effect	.0341***	.0067	.1533	[.0211,.0472]^a^
Total indirect	.0577	.0054	.2591	[.2118,.3035]
Ind1: PCE-R → PSS → LS	.0333	.0050	.1494	[.1054,.1940]
Ind2: PCE-R → ML → LS	.0115	.0025	.0517	[.0309,.0754]
Ind3: PCE-R → PSS → ML → LS	.0129	.0023	.0580	[.0392,.0787]

All models included the seven demographic covariates; 5,000 bootstrap resamples. β (cs) = completely standardized effect. ^a^CIs for total and direct effects are unstandardized (B-metric). All three specific indirect pathways remained significant, and Ind1 remained the largest specific pathway by point estimate, reproducing the substantive pattern of the primary model. ***p < .001.

Second, the model was re-estimated with Presence of Meaning (PM) and Search for Meaning (SM) separately specified as the second mediator in turn (**Table 8**). All three indirect pathways were significant in both models. Pathways through PM [Ind2 = .074, 95% CI (.049,.103); Ind3 = .082, 95% CI (.060,.107)] were larger than those through SM [Ind2 = .023, 95% CI (.010,.039); Ind3 = .029, 95% CI (.015,.045)], indicating that the mediating role of ML was principally, though not exclusively, attributable to the presence dimension. Notably, in the PM model the contrast between Ind1 and the serial pathway (C2) did not reach significance, indicating comparable magnitudes—a pattern consistent with the dual-source equivalence observed in the primary model (C3). The main conclusions were fully robust to the composite operationalization.

**Table 8:** Sensitivity analyses specifying MLQ subscales as the second mediator.

Effect	PM model β (cs)	95% CI	SM model β (cs)	95% CI
Total effect	.4142	—	.4142	—
Direct effect	.1171***	—	.1685***	—
Ind1: PCEs → PSS → LS	.1407	[.0930,.1868]	.1942	[.1456,.2420]
Ind2: PCEs → PM/SM → LS	.0741	[.0485,.1031]	.0227	[.0096,.0389]
Ind3: PCEs → PSS → PM/SM → LS	.0823	[.0595,.1072]	.0288	[.0150,.0449]
C1: Ind1 − Ind2	.0666	[.0081,.1233]	.1715	[.1171,.2254]
C2: Ind1 − Ind3	.0584	[−.0015,.1140]	.1654	[.1125,.2176]
C3: Ind2 − Ind3	−.0082	[−.0452,.0279]	−.0061	[−.0243,.0103]

PM, Presence of Meaning; SM, Search for Meaning. Both models included all seven covariates; 5,000 bootstrap resamples; completely standardized effects. All specific indirect pathways were significant in both models. In the PM model, the contrast between Ind1 and the serial pathway (C2) did not reach significance, indicating comparable magnitudes of these two pathways when presence of meaning alone serves as the second mediator. ***p < .001.

Third, paternal and maternal education were combined into a single parental education covariate; as reported in the **Table 5** footnote, the combined indicator was non-significant in all three equations (all *p* >.47) and all indirect effect estimates were essentially unchanged (Ind1 = .162, Ind2 = .052, Ind3 = .063), confirming that the opposing coefficient signs in the primary model reflect a suppression artifact with no bearing on the core conclusions.

Fourth, the model was estimated separately for male (*n* = 439) and female (*n* = 913) participants (**Table 9**). All three indirect pathways were significant in both groups, confirming that the serial mediation structure replicated across genders. One path differed significantly between groups: the PCEs–PSS association was stronger among males (*z* = 3.09, *p* = .002). Two further paths showed marginal differences in opposite directions—PSS → LS was marginally stronger among males (*p* = .082) and ML → LS marginally stronger among females (*p* = .059)—and the direct PCEs–LS association reached significance among females but not males. These patterns are discussed further in Section 4.4.

**Table 9:** Serial mediation model by gender.

Panel A. Path coefficients and between-group comparisons				
Path	Male (n = 439) B (SE)	Female (n = 913) B (SE)	z	P
PCEs → PSS	.741 (.034)	.609 (.025)	3.09	.002
PCEs → ML	.456 (.098)	.316 (.067)	1.17	.240
PSS → ML	.690 (.095)	.607 (.070)	0.70	.483
PSS → LS	.307 (.052)	.195 (.039)	1.74	.082
ML → LS	.098 (.025)	.155 (.018)	−1.89	.059
PCEs → LS (direct)	.083 (.051), *p* = .108	.159 (.036)***	−1.20	.229
Panel B. Indirect effects (completely standardized)				
Effect	Male	95% CI	Female	95% CI
Ind1	.2579	[.1704,.3480]	.1212	[.0645,.1770]
Ind2	.0506	[.0162,.0919]	.0502	[.0238,.0807]
Ind3	.0567	[.0239,.0947]	.0587	[.0350,.0858]

Both group models controlled for age, household registration, number of siblings, paternal and maternal education, and family income; 5,000 bootstrap resamples. Between-group differences were tested as z = (B_1_ − B_2_)/√(SE_1_² + SE_2_²). All three specific indirect pathways were significant in both groups. ***p < .001.

## 4 Discussion

### 4.1 Summary of main findings

The present study investigated psychological mechanisms linking positive childhood experiences (PCEs) to life satisfaction among 1,352 Chinese college students, proposing perceived social support and meaning in life as sequential mediating resources within an integrated Broaden-and-Build and Conservation of Resources framework. Four principal findings emerged. PCEs were positively and significantly associated with life satisfaction (completely standardized total association = .414), supporting H1. All three specific indirect pathways were statistically significant, collectively accounting for approximately 66.5% of the total PCE–life satisfaction association. The independent mediation of perceived social support (Ind1 = .161) emerged as the largest single indirect pathway, exceeding both the independent mediation of meaning in life (Ind2 = .053) and the serial pathway through both mediators (Ind3 = .062), supporting H2. The significant independent mediation of meaning in life (Ind2 = .053, 95% CI [.031,.077]) further confirms that ML functions as a meaningful independent psychological mechanism linking PCEs to life satisfaction, supporting H3. The serial indirect pathway was significant, supporting H4. The direct association between PCEs and life satisfaction remained significant after controlling for both mediators (β = .139), indicating partial rather than complete mediation. A noteworthy feature is that Ind2 and Ind3 did not differ significantly in magnitude (C3: 95% CI [−.036,.019]), a pattern with specific theoretical implications addressed below.

### 4.2 Theoretical implications

#### 4.2.1 Positive childhood experiences and life satisfaction: a resource accumulation perspective

The positive association between PCEs and LS is consistent with evidence that the cumulative quality of early interpersonal environments shows enduring associations with adult well-being (2). Within the Broaden-and-Build framework (11), sustained positive relational experiences during childhood build durable personal resources that persist into adulthood and shape well-being appraisals long after the original experiences have passed. The mediation results extend this theoretical account: PSS and ML together transmitted approximately two-thirds of the total PCE–LS association, partially decomposing the developmental legacy of early positive experiences into identifiable resource-based pathways. This decomposition is consistent with the systematic review of Cunha et al. (4), which documented positive associations between PCEs and LS across 87 studies while noting that PCEs function partly as independent protective factors and partly through mediating psychological resources. Samji et al. (5) further reported, in a population-based study of pandemic-era adolescents in British Columbia, Canada, that effect sizes for PCEs exceeded those for ACEs in relation to depression, mental well-being, and life satisfaction in that sample, supporting the conclusion that positive early experiences constitute an active resource base rather than merely the absence of adversity. From a Conservation of Resources perspective (12, 51), individuals who begin adulthood with richer positive relational histories are better positioned to perceive social support and accrue higher-order existential assets ——a pattern consistent with the resource gain spirals posited by COR theory. Consistent with this framework, latent profile analyses of Chinese young adults found that profiles characterized by high benevolent childhood experiences and low adversity were associated with significantly better LS relative to profiles marked by elevated adversity, providing direct Chinese-context evidence for the resource accumulation value of positive early experiences (6).

#### 4.2.2 Perceived social support as the primary transmission mechanism

The identification of PSS as the dominant mediating pathway (Ind1 = .161, approximately 38.9% of the total association) warrants specific theoretical consideration. From an attachment theory perspective (13), early experiences of family warmth, peer acceptance, and the availability of caring non-parental adults may be internalized as generalized relational schemas, stable representations of others as reliable and supportive, that shape how individuals interpret social cues in adulthood. Individuals whose childhood relational environments provided consistent experiences of being valued may, as young adults, perceive available support more readily, not because their objective networks are necessarily more extensive, but because their relational schemas facilitate more favorable appraisals of social availability. The subjective sense of being supported and embedded in caring relationships then independently contributes to favorable life evaluations through both stress-buffering and main-effect pathways (18). Parallel structural evidence was provided by Yang et al. (21), who found that PSS occupied the upstream mediating position in the chain linking ACEs to LS in a large Chinese adult sample, directly paralleling the structural role of PSS in the present model. Among Chinese college students, Nie et al. (20) found that PSS was positively associated with LS both directly and through sequential mediation involving physical activity and self-control, underscoring the multifunctional resource-mobilizing role of social support. The relative prominence of Ind1 may also reflect population-specific features: for students from rural and economically modest backgrounds navigating an unfamiliar university environment, the subjective sense of being supported by significant others, family, and friends may carry particular salience as a determinant of LS. This contextual interpretation is tentative and requires examination through designs stratified by socioeconomic background and urban-rural origin. The robustness of this pattern is reinforced by the sensitivity analysis excluding PCE items with direct interpersonal-support content (Section 3.3): all three indirect pathways remained significant with the reduced scale, and PSS remained the largest specific pathway by point estimate, indicating that the prominence of the support pathway is not an artifact of item content overlap between the two measures.

#### 4.2.3 The serial pathway: social support as a relational foundation for meaning construction

The significant serial indirect effect (Ind3: completely standardized effect = .062, 95% CI [.042,.085]) is consistent with PSS relating to LS partly through its link to ML. The subjective experience of being reliably supported by significant others constitutes a relational foundation for existential meaning: social embeddedness affirms that one matters to others and belongs to something beyond oneself, experiences identified as phenomenologically central to a meaningful life (31). This account is particularly resonant within the Chinese cultural context, where meaning-making is substantially grounded in occupying a valued role within interpersonal networks (32). A meta-analysis of 135 studies and 397 effect sizes confirmed a significant positive correlation between social support and ML across diverse populations, with subjective social support demonstrating the strongest association among all support subtypes (34). Among Chinese university students, social support negatively predicted maladaptive disengagement behavior through sequential mediation of ML and psychological resilience, with the full mediating chain accounting for 70.2% of the total effect, providing direct evidence for PSS as an upstream antecedent of ML (35). Tian et al. (36) provided longitudinal evidence that PSS predicted depression in college students through chain mediation of gratitude and ML, establishing prospective support for ML as a downstream outcome of PSS processes. Among Chinese adolescents, PSS was associated with greater ML through reduced psychological insecurity and enhanced hope, optimism, and self-efficacy (37).

The finding that the PSS → ML path coefficient (β = .366) substantially exceeded the PCEs → ML coefficient (β = .208) when both were modeled simultaneously suggests that PSS may serve as an amplifying conduit through which early positive relational experiences become associated with a sense of life purpose. This interpretation gains further specificity from the cultural account developed in Section 1.2.4: in a relational cultural context where perceived support constitutes ongoing confirmation of one’s valued standing within the social order, the meaning-conferring function of social support is expected to be particularly pronounced. Consistent with this account, the two MLQ dimensions were strongly and positively correlated in the present sample (r = .716), and search for meaning related positively to life satisfaction (r = .336)—a “harmonious” rather than “opposed” configuration that mirrors the pattern documented in interdependent cultures (46).

The non-significant contrast between Ind2 and Ind3 (C3: B = −.009, 95% CI [−.036,.019]) carries theoretical implications that merit elaboration. This equivalence is consistent with a dual-source account of meaning in life: existential meaning appears to draw, in approximately equal measure, on an early-established foundation associated with the relational content of childhood experience, and on an ongoing stream nourished by currently perceived social support. Meaning, on this account, possesses both developmental sedimentation and present-day plasticity. If corroborated by longitudinal evidence, this pattern would carry a consequential implication for intervention design: limited early relational resources need not foreclose the development of meaning, because strengthening perceived social support in emerging adulthood may offer a compensatory route of approximately comparable magnitude to the early-established pathway. Notably, this equivalence was reproduced in the sensitivity analysis specifying presence of meaning alone as the second mediator (**Table 8**), indicating that it is not an artifact of the composite operationalization. Whether the relative weights of the two sources vary as a function of cumulative adversity exposure remains an open question for the moderated-mediation designs outlined below.

#### 4.2.4 The residual direct association and partial mediation

The persistence of a significant direct association between PCEs and LS after controlling for both mediators (β = .139) indicates that the present model does not fully account for the developmental legacy of positive childhood experiences. Among plausible additional mechanisms, positive affect memory may sustain a favorable affective orientation toward life independently of current social and existential resources (52), and dispositional optimism cultivated through positive early relational environments may independently contribute to favorable life evaluations (53). Yan and Wu (54) proposed an integrated framework in which childhood experiences are associated with stress resilience through the hypothalamic-pituitary-adrenal axis stress response system, suggesting that stress resilience constitutes a further resource-based pathway through which PCEs may relate to adult well-being independently of the social and existential mechanisms captured in the present model. Chen and Tian (55) further revealed that parent-child closeness functions as the core connecting factor between childhood victimization and adulthood well-being, suggesting that early parent-child relationship quality may carry direct well-being associations operating partly independently of PSS or ML in adulthood. The residual direct association thus serves as an empirical prompt for subsequent investigations to articulate and test additional resource-based pathways within the PCE–LS nomological network.

### 4.3 Practical implications

Given the cross-sectional design, the following directions represent theoretically informed hypotheses rather than empirically validated recommendations. At the developmental level, the findings support policies that systematically enrich family relationships, peer environments, and community engagement during childhood and adolescence. Thomson et al. (56) demonstrated that improvements in peer belonging and school climate were prospectively associated with positive changes in emotional health across diverse adolescent groups, corroborating the developmental salience of supportive relational environments as a foundation for later well-being.

At the university level, the dominance of the PSS pathway suggests that institutional efforts to strengthen students’ perceived support availability, particularly among rural-origin and economically modest students, represent a practically relevant lever for promoting LS. Peer mentorship schemes constitute one empirically supported mechanism: a systematic review of peer mentoring in the study entry phase concluded that such programs yield favorable effects on social and academic integration, sense of belonging, and emotional outcomes among beginning undergraduates (57). Structured psychosocial programs delivered in higher education settings more broadly have likewise demonstrated meaningful benefits for students’ emotional well-being in meta-analytic work (58). Other commonly proposed measures—faculty advising programs and structured social engagement activities—remain, at present, theoretical inferences awaiting validation through experimental or quasi-experimental designs.

At the individual level, meaning-focused psychological approaches merit consideration. A quasi-experimental study demonstrated that a 12-session mindfulness program significantly enhanced both PSS and LS with large effect sizes (η = 0.704 and η = 0.510, respectively), indicating that structured group-based interventions can simultaneously address relational and existential well-being resources (59). Integrated interventions targeting relational connection and existential purpose concurrently may be more effective than approaches addressing either dimension in isolation.

### 4.4 Limitations and future directions

Several limitations should be acknowledged. Most fundamentally, the cross-sectional design precludes causal inference: reverse and reciprocal interpretations cannot be excluded on statistical grounds, and the serial mediation results reflect statistical consistency rather than demonstrated temporal ordering. Harman’s single-factor test and the severely degraded fit of the single-factor measurement model both indicate that common method bias is unlikely to account for the observed associations, though these indicators remain preliminary (48). The retrospective assessment of PCEs introduces the potential for mood-congruent recall bias, whereby current well-being may systematically color recollections of childhood experiences, potentially inflating PCE–outcome correlations and partly accounting for the residual direct PCEs–LS association (60, 61). Generalizability is further constrained by the geographic restriction to four universities in Sichuan Province, the focus on tertiary-educated emerging adults, and the convenience sampling procedure, which introduces potential self-selection bias. With respect to gender, multi-group analyses confirmed that the serial mediation structure replicated across males and females; nevertheless, the PCEs–PSS association was significantly stronger among males (z = 3.09, p = .002), and marginal differences suggested that the support pathway may weigh more heavily for males while the meaning pathway may weigh more heavily for females (ps = .082 and.059, respectively; 21), warranting confirmation in gender-balanced samples with formal moderated-mediation designs. The composite operationalization of ML, while supported by the strong inter-subscale correlation (r = .716) and robust to subscale-level sensitivity analyses, may nonetheless obscure differential contributions of meaning presence and search that carry distinct theoretical implications for well-being (22, 46). Although discriminant validity analyses and item-removal sensitivity analyses converged in supporting the empirical distinguishability of PCEs and PSS, their partial content overlap cannot be fully eliminated statistically; latent variable modeling with method-variance controls is recommended for future replication. Finally, the moderating role of PCEs on ACE outcomes yields inconsistent results across studies (4), underscoring that boundary conditions including developmental timing, adversity severity, and cultural context merit systematic examination.

Future research should employ longitudinal designs with multiple assessment waves to establish temporal precedence and clarify whether the residual direct PCEs–LS association reflects a genuine unmediated pathway or the operation of unmeasured distal mechanisms (e.g., random-intercept cross-lagged panel modeling; 62). Modelling Presence of Meaning and Search for Meaning as distinct mediators would yield greater theoretical precision regarding their differential antecedents and associations with LS (46). Incorporating additional psychological resources—including resilience, self-compassion, optimism, and emotion regulation—would provide a more comprehensive account of the PCE–LS nomological network; explicit moderated-mediation designs should further examine whether the relative dominance of the PSS pathway varies as a function of socioeconomic background, gender, urban-rural origin, or cumulative adversity exposure. Finally, cross-cultural replication incorporating measurement invariance testing is essential to determine whether the present mediation architecture generalizes beyond collectivist cultural contexts (63).

## 5 Conclusion

The present study indicates that the association between PCEs and LS among Chinese college students is substantially accounted for by two sequentially organized psychological resources, PSS and ML, which together correspond to approximately 66.5% of the total association. The findings are consistent with a resource pathway in which the relational legacy of positive early experiences is reflected in a heightened sense of social support availability in emerging adulthood, which in turn relates to LS both directly and through its association with existential meaning. The comparable magnitudes of the independent ML pathway and the serial pathway further suggest that meaning may draw on two complementary sources—one rooted in the richness of early relational experience and one linked to the ongoing perception of social support—pointing to the multifaceted ways in which childhood relational capital may be connected to evaluative well-being in young adulthood. Although the cross-sectional design precludes causal inference and these interpretations await longitudinal corroboration, the robustness of the observed pattern across measurement operationalizations, item-content specifications, and genders lends confidence to its structural reliability. For Chinese college students—particularly those from rural and economically modest backgrounds, who constitute a substantial yet understudied segment of the undergraduate population—these findings offer a theoretically grounded basis for interventions attentive to both the relational and existential dimensions of well-being. Strengthening students’ perceptions of available social support and fostering a coherent sense of life purpose represent complementary and potentially mutually reinforcing intervention targets that warrant systematic evaluation in future experimental and longitudinal research.

## Data Availability

The raw data supporting the conclusions of this article will be made available by the authors, without undue reservation.
